# Unrecognized grief - Prevalence and comorbidity of prolonged grief among refugees in Sweden

**DOI:** 10.1016/j.jmh.2024.100274

**Published:** 2024-10-12

**Authors:** Anna Leiler, Jennifer Meurling, Elisabet Rondung, Shervin Shahnavaz, Gerhard Andersson, Anna Bjärtå

**Affiliations:** a Mid Sweden University 831 25 Östersund, Sweden; b Karolinska institutet 171 77 Stockholm, Sweden; c Linköping University 581 83 Linköping, Sweden

**Keywords:** Refugees, Mental health, Prolonged grief, Depression, Post-traumatic stress disorder

## Abstract

Refugees often experience multiple losses. Despite this, and even though the loss is often due to unnatural causes and violent killings, the presence of Prolonged Grief (PG) among refugees may be obscured by other diagnoses such as depression and post-traumatic stress disorder (PTSD). In this cross-sectional study, we assessed the prevalence of PG and its comorbidity with depression and PTSD among 679 adult refugees in Sweden. Results showed that 401 (59.06 %) individuals had lost someone close to them, whom they were grieving intensely. Of these, 76 individuals (18.95 % of 401) fulfilled the criteria for PG. In the full sample, 304 individuals fulfilled the criteria for depression and 56 (18.42 % of 304) of these individuals also fulfilled the criteria for PG. Similarly, 315 fulfilled the criteria for PTSD. Among these individuals, 201 reported loss and 64 (20.32 % of 315) also fulfilled the criteria for PG. The results indicate that behind symptoms of depression and PTSD, there may be a layer of grief. This needs to be considered if we are to provide accurate and effective assessments and interventions for refugees.

## Highlights

• Prolonged Grief among refugees may be hidden behind PTSD and depression
• About 20 % of the included refugees with PTSD also experienced Prolonged Grief
• About 20 % of the refugees with depression also suffered from Prolonged grief
• Acknowledging Prolonged Grief is important for adequate assessment and treatment

## Introduction

Loss is an unavoidable part of life. We love, we lose, and mostly, we learn to live with the loss. However, some people experience loss to an unbearable extent. Individuals with a refugee background (further on referred to as *refugees*) are forced to leave their homes due to war, violence or persecution. This process is full of losses: of a country, a culture, and almost inevitably, of loved ones. Family members may be killed, disappeared, or, in good circumstances, forced to live oceans apart. In a study including Syrian refugees in Sweden, 77.6 % reported having experienced forced separation from family or close friends, and 70.9 % reported loss or disappearance of family member(s) or loved one(s) (Sigvardsdotter et al., 2017). Another study reported that 92 % of asylum seekers and refugees had experienced the loss of a loved one (Comtesse and Rosner, 2019). Loss and separation can thus be considered central aspects of the refugee experience. The term Migratory Grief has been used to describe the experience of migratory loss and separation, as well as the psychological distress it can lead to. A recent review has found a link between migratory grief and psychopathology (Renner et al., 2024). Besides loss, the migration process is marked by numerous stressors, including traumatic experiences before the flight (Mesa-Vieira et al., 2022) and post-migratory stressors occurring after arriving to a new country (Gleeson et al., 2020), not to mention the often hazardous flight (United Nations High Commissioner for Refugees 2015). Unsurprisingly, high levels of distress have been documented in several systematic reviews (Blackmore et al., 2020; Mesa-Vieira et al., 2022). Although there are exemptions (see for example Hajak et al., 2021), focus is usually on depression and Post Traumatic Stress Disorder (PTSD), potentially overlooking loss and grief.

Normally, when a loss is expected and the grief uncomplicated, grieving can be a healthy process, after which the bereaved maintains an attachment to the deceased while accepting to live on without them (Shear, 2012). This process often works, and resilience is the most common response to bereavement (Bonanno, 2005). There are, however, factors that complicate this process and hinder natural recovery. One such factor regards the circumstances of the death, for example, if the death was caused by violent killings (Djelantik et al., 2020). Social constraints are also associated with impaired adjustment following bereavement (Juth et al., 2015). In recognition of the cases where the mourning goes awry, in 2018, Prolonged Grief Disorder (PGD) was added as a diagnosis in the 11th revision of the International Statistical Classification of Diseases and Related Health Problems (ICD-11, World Health Organization 2019). In 2022, it entered the text-revised version of the fifth Diagnostic and Statistical Manual of Mental Disorders (DSM-5-TR, American Psychiatric Association 2022). In both manuals, the diagnosis requires the death of a loved one, separation distress in terms of a persistent grief response, intense emotional pain and functional impairments. Both manuals distinguish PGD against other conditions. In ICD-11, a diagnosis can be set six months after the death, whereas in DSM-5-TR, more than 12 months must have passed. Both manuals stresses that cultural customs related to grief and mourning must be taken into consideration.

Some symptoms of PGD overlap with depression and PTSD, but not all. For example, sadness is a core symptom both in depression and PGD, whereas yearning is a core symptom in PGD but not in depression. Both PTSD and PGD include intrusive thoughts and images (in PGD focused on the deceased). In PGD, this is often paired with behaviors intending to render a sense of proximity to the deceased, which is usually not seen in association with PTSD (see Shear, 2015, for a comparison of the symptoms of PGD, depression, and PTSD). The occurrence of comorbid PGD is important to acknowledge, since PGD is associated with impairments in both mental and physical health (Prigerson et al., 1997; Thimm et al., 2020) and individuals with PGD tend to have more severe mental health problems overall (see for example Lechner-Meichsner et al., 2024).

The prevalence of PGD among bereaved in the general population has been estimated to be 3.3 % - 4.2 % (Rosner et al., 2021). This prevalence is elevated in vulnerable subgroups, and among refugees, a pooled prevalence of 33.2 % (Kokou-Kpolou et al., 2020) has been found. A German study including asylum seekers and refugees found that 20 % met the criteria for PGD and 16 % the criteria for DSM-5 persistent complex bereavement disorder. High rates of probable PTSD (45 %) and depression (42 %) were also noted (Comtesse and Rosner, 2019) . PGD clearly seems to be a significant cause of distress among refugees.

Comorbidity between PGD, PTSD and depression is high: it has been suggested that 63 % of individuals with symptoms of PGD have symptoms of depression, and that 49 % also have symptoms of PTSD (Komischke-Konnerup et al., 2021). In this regard, refugees are no exception. In a review on bereavement among adult refugees, significant associations between PGD and PTSD, and between PGD and depression, were found (Kokou-Kpolou et al., 2020). Studies have identified different symptom clusters among affected individuals. For example, a study on Iraqi internally displaced persons identified four classes: low-symptoms 17.6 %, PGD 33.7 %, PTSD 12.1 %, and comorbid PTSD+PGD 36.7 % (Jann et al., 2024). Another study including asylum seekers in Germany found three clusters: PGD 30 %, PGD+PTSD 32 %, and resilient 38 % (Comtesse et al., 2024). Predictors of these clusters included the sudden or violent death of a loved one, which was a distinguishing factor for PTSD, and insecure residence status, which predicted membership in the PGD and PGD+PTSD clusters. Higher attachment anxiety was also associated with the PGD+PTSD cluster (Comtesse et al., 2024).

The high levels of comorbidity, combined with a limited focus on PGD, may imply that among refugees diagnosed with PTSD and depression, there may be individuals with unrecognized PGD. In effect, they may present with symptoms that are not addressed in interventions focused on PTSD and depression. Including questions on experiences of loss and grief-related symptoms in the assessment of mental health among refugees may render a more nuanced understanding of the complex needs of the population. This may pave the way for better adapted and culturally sensitive interventions. To shed more light on this new diagnosis, we report on the prevalence of Prolonged Grief (PG) symptoms, as well as on its comorbidity with symptoms of PTSD and depression among refugees in Sweden.

## Material and methods

As part of a larger project aiming to develop online psychological assessment and intervention tools for refugees (see Meurling et al., 2023) we conducted a cross-sectional survey. Data were collected in Sweden between May and September 2020 via an online questionnaire. The study has been reviewed and approved by the Swedish Ethical Review Authority (2020–00214).

### Participants and recruitment

In brief, we recruited participants via social media, education centers, an asylum housing and other meeting points for refugees. Inclusion criteria were literacy in Arabic, Dari, Farsi, English or Swedish, minimum age 18, and having refugee background. Refugee background was self-defined independent of legal status: all participants answered a question on whether they had fled their homes due to war, conflict, or persecution. A convenience sample of 823 respondents from all regions in Sweden was recruited. All participants provided their informed consent. For further details regarding recruitment, see Meurling et al. (2023). Of the 823 participants, we excluded 64 individuals without refugee background, 25 under the age of 18 years, and 55 due to missing data. The final sample thus consisted of 679 individuals. Of these, 401 (59.06 %) reported that they had lost someone close to them, whom they were grieving intensely. The mean age in the full sample was 32.76 years (*SD* 10.67) and on average, the participants had been in Sweden for 4.67 years (0–11 years). See Table 1 for further demographic information.

**Table 1:** Participant characteristics.

Variable	Full sample (*n* = 679)	Bereaved subgroup (n = 401)
	n (%)	n (%)
*Sex*		
Man	437 (64.36)	271 (67.58)
Woman	239 (35.20)	129 (32.17)
Other	3 (0.44)	1 (0.25)
*Nationality*		
Syria	237 (34.90)	152 (37.91)
Afghanistan	173 (25.48)	86 (21.45)
Palestine	41 (6.04)	26 (6.48)
Iran	34 (5.01)	13 (3.24)
Stateless	31 (4.57)	20 (4.99)
Iraq	30 (4.42)	21 (5.24)
Eritrea	24 (3.53)	17 (4.24)
Yemen	19 (2.80)	16 (3.99)
Somalia	15 (2.21)	–
Other	75 (11.05)	50 (12.47)
*Residence permit*		
No	168 (24.74)	110 (27.43)
Temporary	185 (27.25)	109 (27.18)
Permanent	326 (48.01)	182 (45.39)
*Marital status*		
Single	298 (43.89)	166 (41.40)
Married/cohabiter	293 (43.15)	177 (44.14)
Divorced/separated	61 (8.98)	38 (9.48)
Widow/widower	3 (0.44)	3 (0.75)
Unknown	24 (3.53)	17 (4.24)

*Note.* Other *=* 10 or fewer participants

### Instruments

To investigate the experience of loss we formulated the following question: Have you lost a loved one, whom you are grieving intensely? yes/no. This question served as a gateway, only individuals with a positive answer were forwarded to the assessment of PG.

Symptoms of PG was assessed with the Prolonged Grief Disorder -13 scale (PG-13, Prigerson et al., 2009). Two of its 13 items, item 3 on duration and 13 on impairment, are answered “yes” or “no” and the remaining 11 symptom items are scored on a 5-point scale (either by frequency, ranging from “not at all” to “several times a day”, or by intensity, ranging from “not at all” to “overwhelmingly”). A preliminary diagnosis of PG is set using a diagnostic algorithm including criterion A; the experience of loss (here “yes” on the question about loss), B; separation distress (daily grief related yearning, item 1 and 2), C; duration longer than 6 months (“yes” on item 3), criterion D; cognitive, emotional and behavioral symptoms at least “once a day” or “quite a bit” (item 4–12), and E; the grief causes significant impairment (“yes” on item 13). PG-13 total scores range from 11–55, however, there are currently no official cutoffs for severity levels. PG-13 has previously been used in various cultural settings and with refugee populations (Işıklı et al., 2022; Lacour et al., 2020). Cronbach's α in the present sample was 0.91.

Depressive symptoms were measured with the Patient Health Questionnaire- 9 (PHQ-9) (Kroenke and Spitzer, 2002). It has nine items, each scored from 0–3, total score 27. A diagnosis is made if five or more of the nine symptoms are scored 2 or above, and one of these is anhedonia or depressed mood. This algorithm was used as a proxy for a depression diagnosis. Cronbach's α in the present sample was 0.90.

Symptoms of PTSD was assessed with the PTSD Checklist for DSM-5 (PCL-5) (Blevins et al., 2015). The PCL-5 has 20 items and is scored from 0 to 4, total score 80. Although several cutoffs have been suggested (Zuromski et al., 2019), we here chose the more conservative cutoff at 38 as proxy for PTSD diagnosis. Cronbach's α in the present sample was 0.96

The complete material was translated into Arabic, Dari, Farsi, English, and Swedish. For the current study, we translated PG-13 to Dari and Farsi, PCL-5 to Dari, and the item on loss (*Have you lost someone close to you, whom you are grieving intensely?*) to all target languages. Remaining instruments either had available translations or had been translated and validated by us in previous projects (Leiler et al., 2019; Bjärtå et al., 2018). Instruments were selected based on cross-cultural validity, brevity, and / or previous use in refugee populations. For more details on instruments and translation, see Meurling et al. (2023).

Since this study was conducted during the COVID-19-pandemic, we added questions about the pandemic in order to analyze if it affected mental health outcomes. We asked about respondents’ knowledge of the pandemic and the degree to which it had affected them negatively (1 = Not at all – 5 = Extremely).

### Analyses

Prevalence and comorbidity of symptoms of PG, PTSD, and depression were analyzed descriptively using frequencies and percentages. Pearson's Chi-square was used to examine differences in depression and PTSD among those who had and had not experienced loss. Differences in mean values were assessed using *t*-tests. We also present descriptives of the COVID-19 items and the associations to the mental health outcomes (symptom scores). All analyses were made with SPSS (version 29).

## Results

Of the 679 participants, 401 (59.06 %) reported having lost someone close to them, whom they were grieving intensely. Of these 401 bereaved individuals, 76 individuals (18.95 %) fulfilled the criteria for PG as assessed by PG13 (see Table 2), with a mean score of 32.07 (*Sd* 11.05). There were no sex differences, *t*(398) = 0.90, *p* = .93, *d* = 0.01 (Δ = 0.11, 95 % CI [-2.22, 2.44]).

**Table 2:** Prevalence and comorbidity of PG, PTSD, and depression among those bereaved and not, as well as in the full sample.

Variable	n ( %)			
	Bereaved	Not bereaved	Total	
TOTAL	401 (59.06)	278 (40.94)	679 (100)	Chi-square
PG	76 (18.95)	NA	76 (11.19)	
Only PG, no comorbid condition	7 (1.75)	NA		
Depression	191 (47.63)	113 (40.65)	304 (44.77)	3.24, *p* = .07
Only depression, no comorbid condition	31 (7.73)	24 (8.63)	55 (8.10)	0.26, *p* = .61
PTSD	201 (50.12)	114 (41.01)	315 (46.39)	5.49, *p* = .019
Only PTSD, no comorbid condition	33 (8.23)	25 (8.99)	58 (8.54)	0.04, *p* = .84
Comorbid PG, depression & PTSD	51 (12.72)	NA		
Comorbid PG & depression, no PTSD	5 (1.25)	NA		
Comorbid PG & PTSD, no depression	13 (3.24)	NA		
Comorbid PTSD & depression, no PG	104 (25.94)	89 (32.01)	193 (28.42)	2.98, *p = .*084

*Note.* PG = Symptoms of Prolonged Grief, PTSD = Symptoms of Post Traumatic Stress Disorder

**Fig. 1:**
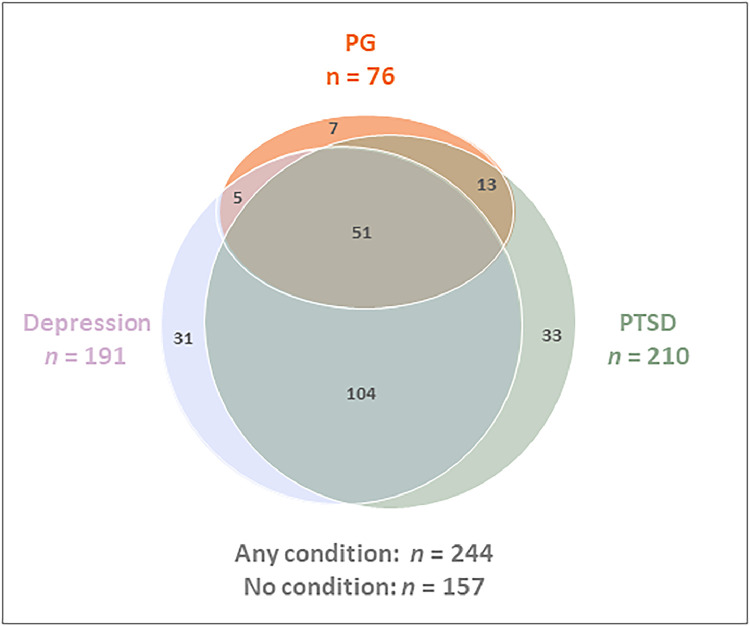
Venn diagram showing potential cases of PG, Depression, and PTSD among those bereaved (n = 401).

**Fig. 2:**
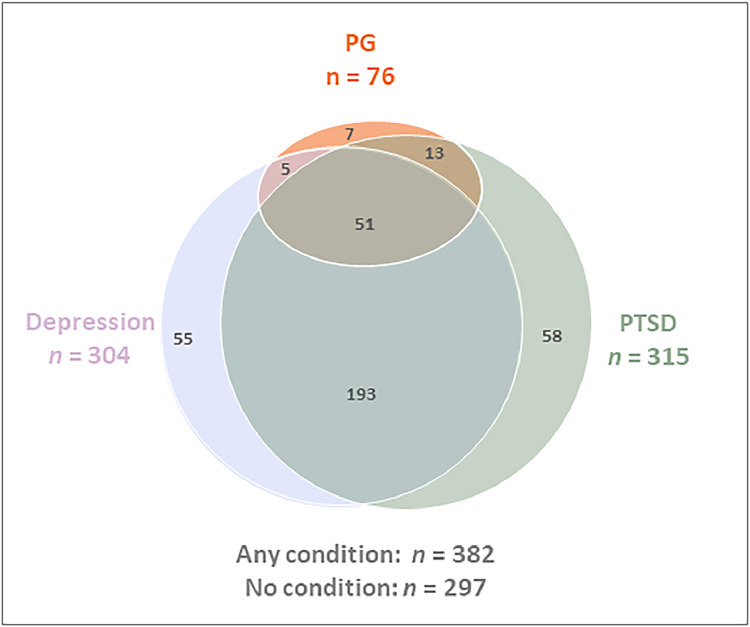
Venn diagram showing potential cases of PG, Depression, and PTSD in the full sample (n = 679).

Of the full sample (679 individuals) 304 (44.77 %) fulfilled the criteria for depression (*M*_PHQ_ = 12.97, *SD* 7.24), and 315 (46.39 %) for PTSD (*M*_PCL-5_ = 36.07, *SD* 20.43). Of the 304 participants classified with depression, 191 reported loss and 56 (18.42 % of 304) also fulfilled the criteria for PG. Similarly, among the 315 classified as having PTSD, 201 reported loss and 64 (20.32 % of 315) also fulfilled the criteria for PG. A total of 51 participants exhibited comorbid symptoms of all three conditions. See Fig. 1, Fig. 2 for an illustration of the overlap between conditions in the bereaved sub-sample and in the full sample.

Mean scores on the PHQ-9 did not differ between those who had and had not experienced loss. However, the mean scores on PCL-5 were higher among those who had experienced loss (*M* 38.31, *SD* 19.99) compared to those who had not (*M* 32.83 (*SD* 20.67), *t*(677) = 3.46, *p* < .001, *d* = 0.27 (Δ = 5.48, 95 % CI [2.38, 8.59]).

Of all participants, the vast majority (98.20 %) had knowledge of the COVID-19 pandemic and were to a large degree negatively affected by the situation, with 71.21 % answering moderately or above (≤ 3). Bereaved participants (*n* = 396) who were more negatively affected by the pandemic reported more symptoms of depression (*r* = 0.163, *p* < .001) and PTSD (*r* = 0.169, *p* < .001). However, being negatively affected by COVID-19 was not significantly correlated with PG-13 symptom scores, *r* = 0.091.

## Discussion

Almost 60 % of the participants reported loss. Of these individuals, almost a fifth fulfilled the criteria for PG, as assessed by PG-13. Although lower than in the study of Sigvardsdotter et al. (2017), the individuals participating in this study had experienced loss to a high extent. Looking at the entire sample, the prevalence of PG was 11.19 %, which is clearly lower than the 33.2 % reported by Kokou-Kpolou et al. (2020), even though this study took place during the Covid-19-pandemic. In addition to potential differences between the samples, one explanation could be that individuals who in our study answered that they had not experienced loss did not answer the questionnaire PG-13. There is a risk that some individuals misunderstood the question regarding loss, and that they would have fulfilled the criteria for PG on the PG-13, if they had answered it. The prevalence in this sample is still far higher than in the general population (Rosner et al., 2021). Of the individuals who had experienced loss, a majority didn't fulfill the criteria for PG. Two implications can be drawn from this: firstly, that resilience, even among these heavily burdened individuals, is still the most common response to bereavement (Bonanno, 2005). A second conclusion is that although it may not be the most common response, a significant proportion of the sample did suffer from PG, which warrants attention. A majority of those who fulfilled the criteria for PG had comorbid depression, PTSD, or both. One-fifth of the individuals who could be classified as having PTSD also fulfilled the criteria for PG, as did nearly as many of the individuals classified as having depression. This indicates that behind these well-acknowledged diagnoses, there may be another, further complicating layer of grief. Interestingly, PTSD was both more common and more severe among those who had experienced loss. PG symptoms have in a review been found to predict symptoms of both PTSD and depression (Janshen and Eisma, 2024) but in the current study, the difference in symptomatology was only seen in PTSD, not in depression. These results point to a need for a deeper understanding of how PG is linked to other conditions such as PTSD and depression, especially in this population.

## Limitations

The results of this study should be viewed in the light of certain limitations. One is the fact that self-report questionnaires were used as proxies for diagnoses, something that has previously been shown to inflate prevalence rates (Henkelmann et al., 2020). Another potentially inflating factor is the context. At the time of data collection, COVID-19 had just been declared a pandemic. Even though prevalence rates of PTSD and depressive symptoms are similar to previous studies (Leiler et al., 2019), we expected data to be affected by the pandemic situation, as confirmed by the correlational analyses. While this association was not found in relation to PG in bereaved participants, loss due to the pandemic may have inflated the frequency of reported loss. Unfortunately, we did not assess the causes of loss. The question on loss was formulated “Have you lost a loved one, whom you are grieving intensely?”. We can thus not be sure that all the individuals who answered “yes” to the loss question had experienced the death of a loved one. This could also differ between translations. In Farsi and Dari, the word chosen for loss, “az dast dadan”,  is mainly used when the loss is due to death.

Generally, when conducting studies including refugees, cultural sensitivity of the used instruments should be considered. Although PG-13 has previously been used in refugee populations (Lacour et al., 2020), it was developed in the US, whereas the participants of this study all came from the Global South. A suggestion for future studies could be to add interviews with the proposed supplementary grief module of the DSM-5 Cultural Formulation Interview (American Psychiatric Association 2013) for increased cultural sensitivity.

Another limitation regarding instruments concerns the fact that we used the first version of the PG-13 scale (Prigerson et al., 2009). This instrument was developed before PGD was included as a diagnosis in ICD-11 and DSM-5-TR and does not fully correspond to the diagnostic criteria of any of the manuals. This is important, since prevalence rates of PG differ depending on the criteria used. Boelen and Lenferink (2020) found a prevalence of 11.2 % when using the criteria in PG-13 (Prigerson et al., 2009) compared to 19.8 % when using the ICD-11 criteria in the same sample. These factors indicate that the prevalence of PG could have been different, had we used for example PG-13-R, which was released after the data collection of the current study (Prigerson et al., 2021). Furthermore, this study is based on an uncontrolled sampling method, limiting its generalizability.

Even so, this study, with its focus on loss and grief, can contribute to an increased understanding of contextual factors that shape refugees’ psychological well-being. Without this, our understanding of the refugee experience risks being erroneous and may result in inaccurate assessments and treatment. We argue that assessing symptoms among refugees ought to involve a nuanced understanding of potential root causes to better address the needs of the population, and to be able to develop tailored and culturally sensitive interventions.

## Funding source

The study was funded by the Swedish Research Council (2018–05827). The funder had no role in the data collection, analysis or reporting of the study findings.

## CRediT authorship contribution statement

**Anna Leiler:** Writing – review & editing, Writing – original draft, Methodology, Investigation, Formal analysis, Data curation, Conceptualization. **Jennifer Meurling:** Writing – review & editing, Resources, Project administration, Methodology, Investigation, Formal analysis, Data curation, Conceptualization. **Elisabet Rondung:** Writing – review & editing, Supervision, Methodology, Investigation, Conceptualization. **Shervin Shahnavaz:** Writing – review & editing, Resources, Methodology, Investigation, Conceptualization. **Gerhard Andersson:** Writing – review & editing, Supervision, Resources, Methodology, Investigation, Funding acquisition, Conceptualization. **Anna Bjärtå:** Writing – review & editing, Supervision, Resources, Project administration, Methodology, Investigation, Funding acquisition, Formal analysis, Data curation, Conceptualization.

## Declaration of competing interest

The authors declare that they have no known competing financial interests or personal relationships that could have appeared to influence the work reported in this paper.

## References

[bib0036] American Psychiatric Association (2013).

[bib0015] American Psychiatric Association (2022).

[bib0033] Bjärtå A., Leiler A., Ekdahl J., Wasteson E. (2018). Assessing severity of psychological distress among refugees with the refugee health screener, 13-item version. J. Nerv. Ment. Dis..

[bib0007] Blackmore R. (2020). The prevalence of mental illness in refugees and asylum seekers: A systematic review and meta-analysis. PLOS Med.

[bib0030] Blevins C.A., Weathers F.W., Davis M.T., Witte T.K., Domino J.L. (2015). The posttraumatic stress disorder checklist for DSM-5 (PCL-5): Development and initial psychometric evaluation. J. Trauma. Stress.

[bib0037] Boelen P.A., Lenferink L.I.M. (2020). Comparison of six proposed diagnostic criteria sets for disturbed grief. Psychiatry Res.

[bib0011] Bonanno G.a (2005). Resilience in the face of loss and potential trauma. Curr. Dir. Psychol. Sci..

[bib0002] Comtesse, H., Rosner, R., “Prolonged grief disorder among asylum seekers in Germany: the influence of losses and residence status,” 10.1080/20008198.2019.1591330, vol. 10, no. 1, Jan. 2019, doi: 10.1080/20008198.2019.1591330. 10.1080/20008198.2019.1591330 PMC6450486 30988893

[bib0024] Comtesse H., Edelhoff H., Rosner R., Lechner-Meichsner F. (2024). Cluster analysis of prolonged grief, posttraumatic stress, and depression symptoms in bereaved asylum seekers and refugees. Eur. J. Psychotraumatol..

[bib0012] Djelantik A.A.A.M.J., Smid G.E., Mroz A., Kleber R.J., Boelen P.A. (2020). The prevalence of prolonged grief disorder in bereaved individuals following unnatural losses: Systematic review and meta regression analysis. J. Affect. Disord..

[bib0005] Gleeson C. (2020). Post-migration factors and mental health outcomes in asylum-seeking and refugee populations: a systematic review. J. Psychotraumatology.

[bib0009] Hajak V.L., Sardana S., Verdeli H., Grimm S. (2021). A systematic review of factors affecting mental health and well-being of asylum seekers and refugees in Germany. Front. Psychiatry.

[bib0035] Henkelmann J.R. (2020). Anxiety, depression and post-traumatic stress disorder in refugees resettling in high-income countries: systematic review and meta-analysis. BJPsych Open.

[bib0027] Işıklı S., Keser E., Prigerson H.G., Maciejewski P.K. (2022). Validation of the prolonged grief scale (PG-13) and investigation of the prevalence and risk factors of prolonged grief disorder in Turkish bereaved samples. Death Stud.

[bib0023] Jann P., Neldner S., Neuner F., Mohammed R. (2024). Complicated grief and posttraumatic stress after loss and separation under terror conditions. J. Trauma. Stress.

[bib0034] Janshen A., Eisma M.C. (2024). Bidirectional associations between prolonged grief symptoms and depressive, anxiety, and posttraumatic stress symptoms: A systematic review. J. Trauma. Stress.

[bib0013] Juth V., Smyth J.M., Carey M.P., Lepore S.J. (2015). Social Constraints are Associated with Negative Psychological and Physical Adjustment in Bereavement. Appl. Psychol. Heal. Well-Being.

[bib0021] Kokou-Kpolou C.K., Moukouta C.S., Masson J., Bernoussi A., Cénat J.M., Bacqué M.F. (2020). Correlates of grief-related disorders and mental health outcomes among adult refugees exposed to trauma and bereavement: A systematic review and future research directions. J. Affect. Disord..

[bib0022] Komischke-Konnerup K.B., Zachariae R., Johannsen M., Nielsen L.D., O'Connor M. (2021). Co-occurrence of prolonged grief symptoms and symptoms of depression, anxiety, and posttraumatic stress in bereaved adults: A systematic review and meta-analysis. J. Affect. Disord. Reports.

[bib0029] Kroenke K., Spitzer R.L. (2002). The PHQ-9: A new depression diagnostic and severity measure. Psychiatr. Ann..

[bib0028] Lacour O. (2020). Prolonged grief disorder among refugees in psychological treatment—association with self-efficacy and emotion regulation. Front. Psychiatry.

[bib0019] Lechner-Meichsner F., Comtesse H., Olk M. (2024). Prevalence, comorbidities, and factors associated with prolonged grief disorder, posttraumatic stress disorder and complex posttraumatic stress disorder in refugees: a systematic review. Conflict Health.

[bib0032] Leiler A., Bjärtå A., Ekdahl J., Wasteson E. (2019). Mental health and quality of life among asylum seekers and refugees living in refugee housing facilities in Sweden. Soc. Psychiatry Psychiatr. Epidemiol..

[bib0004] Mesa-Vieira C. (2022). Mental health of migrants with pre-migration exposure to armed conflict: a systematic review and meta-analysis. Lancet Public Heal.

[bib0008] Mesa-Vieira C. (2022). Mental health of migrants with pre-migration exposure to armed conflict: a systematic review and meta-analysis. Lancet Public Heal.

[bib0025] Meurling J. (2023). An online tiered screening procedure to identify mental health problems among refugees. BMC Psychiatry.

[bib0017] Prigerson H.G. (1997). Traumatic grief as a risk factor for mental and physical morbidity. Am. J. Psychiatry.

[bib0026] Prigerson H.G. (2009). Prolonged grief disorder: psychometric validation of criteria proposed for DSM- V and ICD-11. PLOS Med.

[bib0038] Prigerson H.G., Boelen P.A., Xu J., Smith K.V., Maciejewski P.K. (2021). Validation of the new DSM-5-TR criteria for prolonged grief disorder and the PG-13-Revised (PG-13-R) scale. World Psychiatry.

[bib0003] Renner A., Schmidt V., Kersting A. (2024). Migratory grief: a systematic review. Front. Psychiatry.

[bib0020] Rosner R., Comtesse H., Vogel A., Doering B.K. (2021). Prevalence of prolonged grief disorder. J. Affect. Disord..

[bib0010] Shear M.K. (2012). Grief and mourning gone awry: Pathway and course of complicated grief. Dialogues Clin. Neurosci..

[bib0016] Shear M.K. (2015). Clinical practice. Complicated grief. N. Engl. J. Med..

[bib0001] Sigvardsdotter E. (2017). Development and preliminary validation of refugee trauma history checklist (RTHC)—A brief checklist for survey studies. Int. J. Environ. Res. Public Health.

[bib0018] Thimm J.C., Kristoffersen A.E., Ringberg U. (2020). The prevalence of severe grief reactions after bereavement and their associations with mental health, physical health, and health service utilization: a population-based study. Eur. J. Psychotraumatol..

[bib0006] United Nations High Commissioner for Refugees, “The sea route to Europe: The mediterranean passage in the age of refugees,” Geneva, 2015. [Online]. Available: https://www.unhcr.org/protection/operations/5592bd059/sea-route-europe-mediterranean-passage-age-refugees.html.

[bib0014] World Health Organization, “International statistical classification of diseases and related health problems (11th Revision).” 2019.

[bib0031] Zuromski K.L. (2019). Developing an optimal short-form of the PTSD Checklist for DSM-5 (PCL-5). Wiley Online Libr. Zuromski, B Ustun, I Hwang, TM Keane, BP Marx. MB Stein, RJ Ursano, RC KesslerDepression Anxiety, 2019•Wiley Online Libr.

